# Clinical and radiographic evaluation of local application of insulin versus hyaluronic acid using resorbable collagen sponge in management of periodontal intra-bony defects: a pilot randomized controlled clinical trial

**DOI:** 10.1007/s00784-026-07015-2

**Published:** 2026-07-20

**Authors:** Naglaa El-Wakeel, Nora Abdelgawad, Marwa Nour Eldien

**Affiliations:** 1 Oral Medicine, Periodontology, Oral Diagnosis and Radiology Department, Faculty of Dental Medicine for Girls, Al-Azhar University, Cairo, 11765 Egypt; 2 The Health Insurance Organization of Egypt, Cairo, Egypt

**Keywords:** Hyaluronic acid, Insulin, Infrabony defect, Single flap approach, Periodontal regeneration, Periodontitis

## Abstract

**Objectives:**

We aimed to evaluate the clinical and radiographic effectiveness of locally applied insulin versus hyaluronic acid (HA), each combined with absorbable collagen sponge (ACS), using the single flap approach (SFA) in the management of periodontal intra-bony defects.

**Materials and methods:**

21 periodontitis patients with intra-bony defects were randomly assigned to 3 groups: group I (SFA + insulin + ACS; *n* = 7), group II (SFA + HA + ACS; *n* = 7), and group III (SFA + ACS; *n* = 7). Primary outcome was clinical attachment level (CAL), assessed at baseline and 6 months postoperatively; secondary outcomes included probing pocket depth (PPD), measured at baseline and 6 months; postoperative pain, using visual analogue scale (VAS) at 24 and 48 h; and radiographic parameters, including radiographic linear defect depth (RLDD) and bone density, assessed at baseline and 6 months.

**Results:**

PPD and CAL improved significantly over time in all groups (*P* < 0.001), with no intergroup differences. VAS scores were significantly lower in the insulin and HA groups than in controls at 24 h (*P* = 0.004) and 48 h (*P* = 0.007), with the lowest scores in the insulin group (*P* = 0.008). RLDD decreased significantly in all groups (*P* < 0.001), with a greater reduction in HA + ACS compared to insulin and controls at 6 months (*P* = 0.027). Bone density increased significantly over time in insulin + ACS (*P* < 0.001) and HA + ACS (*P* = 0.022), with no change in the ACS group (*P* = 0.860), with non-significant intergroup differences.

**Conclusion:**

In the management of periodontal intrabony defects, SFA with both insulin and HA soaked in collagen sponge showed favorable clinical and radiographic improvements.

**Clinical relevance:**

Local insulin may represent a promising, simple, and cost-effective biomaterial in periodontal treatment; however, more studies are needed.

**Clinical trial registration:**

NCT05746676.

## Introduction

Periodontitisis one of the most common chronic inflammatory diseases, characterized by progressive destruction of the teeth’s supporting structures. It is caused by interaction between dysbiotic oral microbiota and the host’s inflammatory response, eventually causing tooth loss [1]. Periodontal treatment aims to stop disease progression, along with the removal of different etiologic factors, with an ultimate aim of regenerating the lost periodontal structures [2].

Periodontal regeneration is a complex, multifactorial process that requires coordinated formation of new supporting tissues. Many different regenerating techniques and biomaterials have been investigated over the last few decades, including guided tissue regeneration membranes, bone transplants, and biologically active mediators, either alone or in combinations. A broad category of biologic agents, like growth factors, has progressively gained popularity among clinicians and is routinely used for periodontal regeneration, including autologous blood-derived products and bioactive molecules like enamel matrix derivatives (EMD), bone morphogenic proteins (BMPs), stem cells, and gene therapy vectors. However, the clinical use of many growth factor-based strategies is still limited by being expensive and having a short half-life [3]. This drew interest to other biologically active substances, such as hormones, to help periodontal regeneration [4].

The excellent effects of insulin on soft and hard tissue healing were shown [5]. In soft tissue, insulin enhances healing by increasing the rate of re-epithelialization, angiogenesis, and extracellular matrix secretion by keratinocytes, endothelial cells, and fibroblasts [6]. Further, insulin also has a critical role in bone metabolism and is even considered a bone anabolic agent [7, 8]. Experimental studies suggested that insulin has a significant role in the healing of fractures and peri-implant bone regeneration even in diabetics [9, 10]. Osteoblasts have been found to have functional insulin receptors, and insulin stimulates their growth and collagen formation in vitro. Importantly, after local administration, these effects take place without harmful systemic glycemic alterations [11].

In periodontal therapy, hyaluronic acid (HA) is another biomaterial with excellent biological features. HA has shown beneficial effects as an adjunct to both non-surgical and surgical periodontal therapy [12–14], as well as in periodontal bone and soft tissue regeneration, by reducing inflammation, exerting bacteriostatic, and accelerating wound-healing processes. In the periodontal environment, HA offers both structural and functional benefits. It acts directly by inhibiting the colonization of periodontal pathogens through suppression of microbial proliferation and indirectly supports the process of regeneration by stabilization of granulation tissue and protection of extracellular matrix proteins against degradation. Furthermore, HA was reported to induce osteo-induction and promote the formation of new bone [15, 16].

The single flap approach (SFA) was developed to overcome the drawbacks of the conventional double flap approach, like the post-surgical recession, patient discomfort, and dentin hypersensitivity [17]. Collagen is a highly porous, biocompatible, and biodegradable material that has long been used in dentistry. Collagen sponge was used to fill the extraction socket for stabilization of the blood clot and to reduce postoperative pain, plus offering a favorable environment for the osteoblast’s attachment and proliferation [18, 19].

Collagen has been used for space maintenance in intra-bony defects [20]and in alveolar socket preservation [21, 22]. Further, topical insulin has been proven effective in wound and bone fracture healing, and was found when embedded within atelocollagen hydrogel, to strongly enhance the redifferentiation and production of cartilage matrices in undifferentiated chondrocytes [23, 24]. Moreover, the therapeutic benefits of HA in periodontal regeneration are well known, and collagen sponge was tried before as a scaffold for HA in chondrocyte-mediated contraction and chondrogenesis in vitro, and promising results were obtained [25].

Based on the previously mentioned reports, the clinical evidence on the regenerative potential of insulin in the treatment of periodontal defects is lacking despite promising data. In this work, we thought of using collagen sponge as a slowly degrading, biocompatible, space-maintaining carrier for local delivery of insulin versus hyaluronic acid in the management of periodontal intrabony defects. In this pilot randomized controlled clinical trial, the clinical and radiographic effects of locally applied insulin as a novel biomaterial for periodontal regeneration were assessed, using a single flap approach, versus hyaluronic acid and collagen sponge alone.

## Materials and methods

### Study design and population

**Fig. 1:**
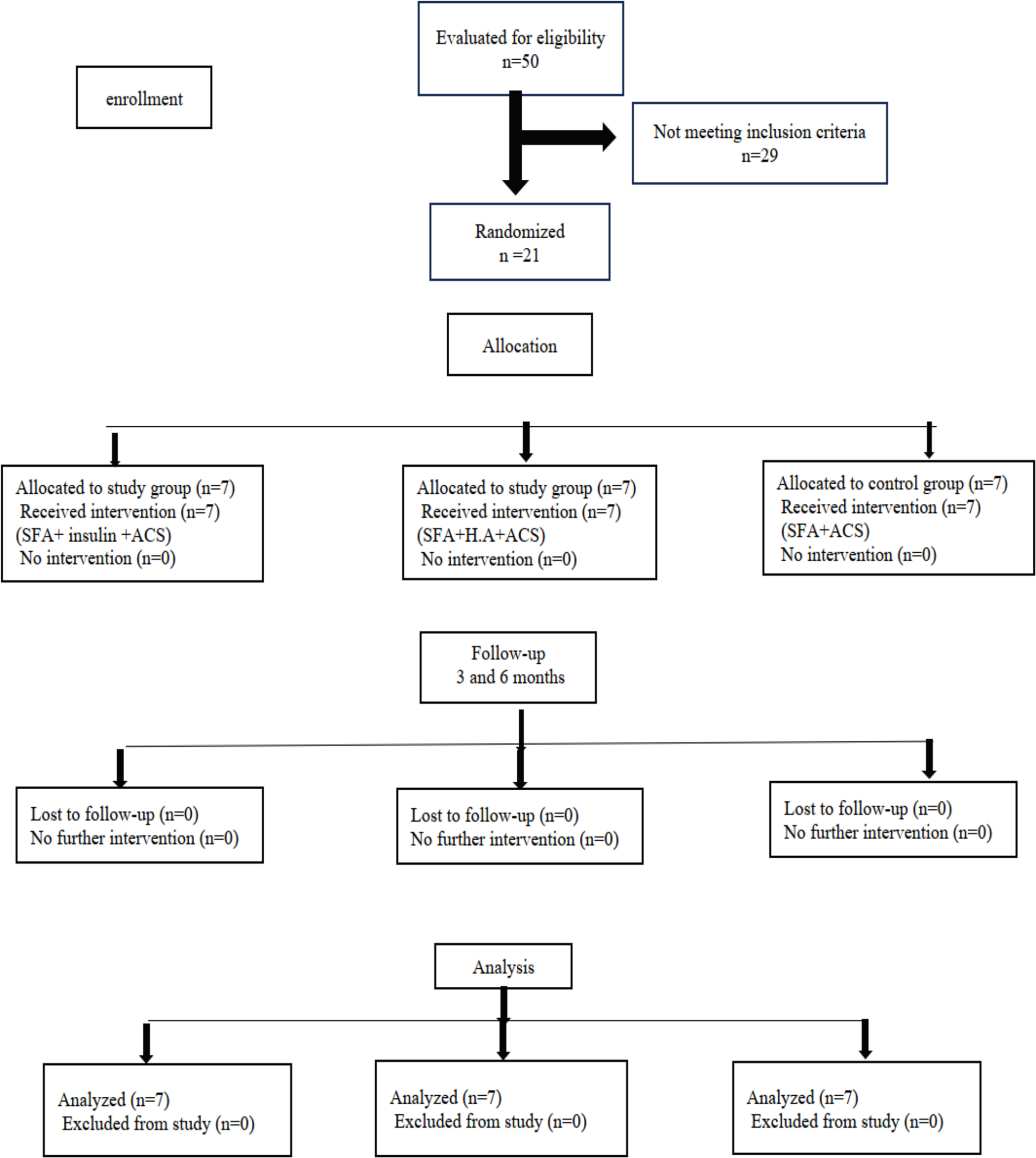
The CONSORT flowchart for this randomized controlled clinical trial

This pilot randomized controlled clinical trial was carried out between May 2023 and May 2025 at the outpatient clinic of Oral Medicine, Periodontology, Oral Diagnosis, and Radiology Department, Faculty of Dental Medicine for Girls, Al-Azhar University, Cairo, Egypt. Patients with periodontitis presenting with CAL loss ≥ 5 mm, PPD ≥ 6 mm, and radiographically confirmed intra-bony defects ≥ 3 mm were considered eligible and equally allocated into one of 3 groups, all treated with open flap debridement via SFA; group I (SFA + ACS+ insulin), group II(SFA + ACS+HA) and group III; (SFA + ACS) Fig. 1. The research protocol was approval from the Institutional Ethics Committee of the Faculty of Dental Medicine for Girls, Al-Azhar University (REC-ME-25-06) and carried out in compliance with the Declaration of Helsinki. The study was registered on ClinicalTrials.gov (NCT05746676), and written informed consent was obtained from all participants before enrollment.

### Inclusion criteria

Participants were ≥ 18 years, systemically healthy, and diagnosed with periodontitis, showing PPD ≥ 6 mm, CAL ≥ 5 mm, persisting 6–8 weeks after non-surgical therapy. Each had at least one intra-bony defect in the maxilla or mandible ≥ 3 mm, involving two- or three-wall defects. When more than one eligible defect per patient was present, the deepest intra-bony defect was selected for inclusion.

### Exclusion criteria

Exclusion criteria were systemic illness potentially affecting healing (e.g., diabetes mellitus, osteoporosis, immunosuppression), antibiotic or anti-inflammatory use within the previous 4 weeks, Smokers, pregnancy or lactation, periodontal surgery in the last 6 months, poor oral hygiene (score 3–6 of oral hygiene index-simplified OHI-S) [26], parafunctional habits, and teeth with furcation involvement or mobility ≥ Grade II.

### Randomization and blinding

The randomization sequence was created using an online tool (www.randomizer.org*)*, and eligible participants were allocated to the study groups in equal ratios (1:1:1). To maintain allocation concealment, an investigator unaffiliated with the trial prepared consecutively numbered and sealed envelopes, (M.N) opened the envelope just before surgery to reveal the treatment assignment. Participants were blinded to the applied material, and outcome assessments were performed by one blinded, experienced investigator (N.A.) who obtained all clinical and radiographic measurements.

### Interventions

#### Preoperative phase

All participants underwent periodontal therapy in accordance with the European Federation of Periodontology (EFP) S3 clinical practice guidelines [1], including step I therapy supragingival plaque control, oral hygiene instructions: twice-daily brushing with a soft toothbrush and rinsing with 0.12% chlorhexidine HCl for two weeks, and patient motivation, along with Step II therapy, consisted of subgingival instrumentation (scaling and root planning). After 6–8 weeks, patients with persistent intrabony defects PPD ≥ 6 mm, CAL ≥ 5 mm, defect depth ≥ 3 mm confirmed radiographically were scheduled for surgical intervention (Step III).

#### Hyaluronic acid preparation

Hyaluronic acid (HA) was prepared at 75 mg/mL by dissolving sodium hyaluronate (800 kDa; HYABLOOM, Bloomage Biotechnology Corp., China) in a solution of phosphate-buffered saline (PBS) and autoclaving for 15 min at 121 °C before use [27].

#### Surgical procedure

**Fig. 2:**
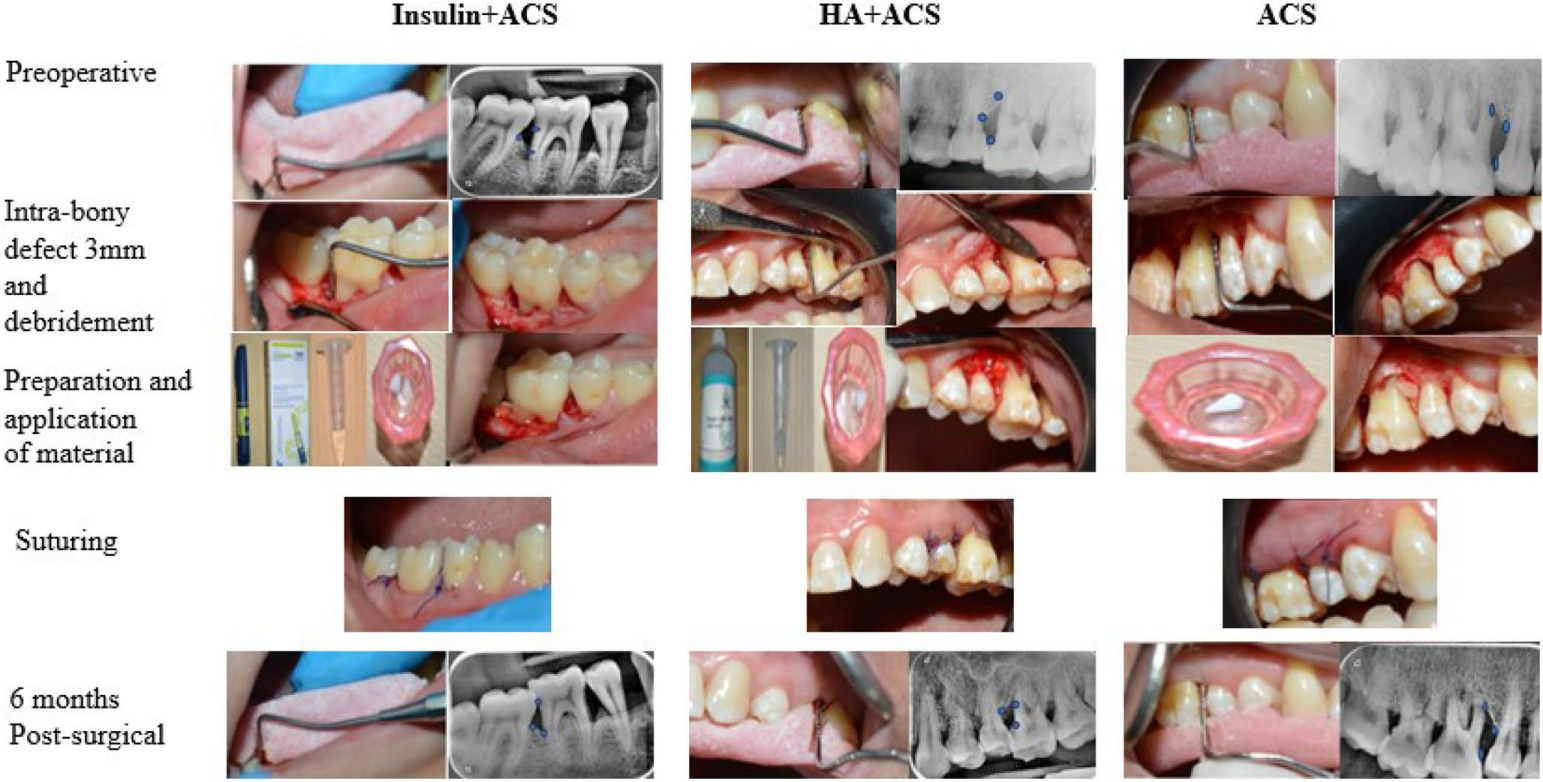
Clinical steps of flap reflection, debridement, preparation, and application of different materials, suturing, and 6 months follow-up for the three studied groups

Under local anesthesia (articaine HCl with 1:100,000 epinephrine; Inibsa Dental S.L.U, Spain, Egypt), intrasulcular incisions were made on the selected tooth and extended one tooth mesially and distally using a 15c blade (KIATO, India). A full-thickness mucoperiosteal flap was elevated using SFA as step III therapy. Granulation tissue was removed using ultrasonic scalers (Woodpecker UDS-K, China) and Gracey curettes (Dentsply, New York, USA), followed by defect inspection and depth recording. Defects in groups I and II were filled with collagen sponges (PARASORB Dental Cones, Resorba Medical GmbH, Germany) trimmed to fit and soaked with insulin solution (20 U/g; insulin degludec, a long-acting insulin analogue; TRESIBA, Novo Nordisk, France) (group I) or ACS soaked in HA solution (group II), while in the control group, defects received collagen sponges alone Fig. 2. Flaps were repositioned without tension and secured with interrupted 4 − 0 absorbable sutures *(*Egysorb, Cairo, Egypt). In group I, blood glucose was measured immediately before and one hour after surgery using a rapid screening device (PERCICHECK, China).

#### Postoperative and follow-up

Patients were instructed to avoid brushing and flossing in the surgical area for 2 weeks, with twice-daily rinsing using 0.12% chlorhexidine. Sutures were removed after 14 days, after which biofilm control was resumed using an ultra-soft toothbrush. Follow-ups were implemented weekly in the first month, at 3 and 6 months.

### Outcome assessment

The primary outcome was changes in CAL, while secondary outcomes were PPD, postoperative pain, radiographic linear defect depth, and bone density.

### Clinical assessment

Clinical parameters included: CAL (measured as the distance from the cemento-enamel junction to the base of the periodontal pocket), and PPD (the distance from the base of the pocket to the gingival margin); all were measured using a William’s periodontal probe, guided by customized acrylic stents with interproximal grooves to ensure accurate postoperative reproducibility. Pain assessment using a visual analogue scale (VAS) as the VAS scale consists of a horizontal line starting from zero to 10, where zero indicates no pain and 10 indicates the intense level of pain, which was done 24 and 48 h postoperatively [28].

### Radiographic assessment

Standardized periapical radiographs were obtained using a PSP sensor (size 2; Durr Dental, Germany) at baseline and 6 months postoperatively (60 kVp, 8 mA, 0.7 mm, 0.10 s) using customized bite blocks and the parallel-angle technique (XCP^®^). The radiographs were digitized and analyzed using DBSWIN 5.7.0 software (Dürr Dental, Germany).

**Fig. 3:**
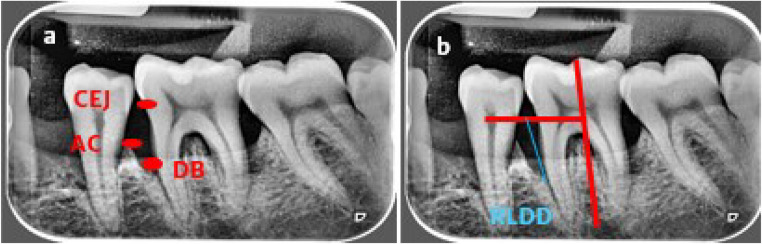
Intrabony defect radiographic measurements. a Reference point identification: cemento-enamel junction (CEJ), alveolar crest (AC), and defect base (DB). b Reference line identification (in red): vertical line corresponding to the long axis and horizontal perpendicular line passing through CEJ and identifying radiographic linear defect depth (RLDD) (in blue)

Radiographic measurements were performed using the cementoenamel junction (CEJ), alveolar crest (AC), and base of the defect (BD) as anatomical landmarks. A line was drawn along the long axis of the tooth, and a second line was drawn perpendicular to it, intersecting the CEJ to provide a consistent reference point for measurements. Radiographic linear defect depth (RLDD) was determined as the distance from this intersection to the base of the defect [29] Fig. 3. Bone density was assessed via grayscale evaluation by averaging three lines on the side of each tooth drawn 0.2 mm apart and parallel to the root surface from the CEJ (as a reference point) to the apex of the root. The mean value of the readings of the three lines was calculated to get the gray level mesial or distal to the tooth for further evaluation. All radiographic measurements were recorded at baseline and 6 months to assess treatment outcomes.

### Statistical analysis

Using IBM SPSS Statistics for Windows, Version 23.0. Armonk, NY: IBM Corp Statistical analysis was performed by checking the distribution of data and using (Kolmogorov-Smirnov and Shapiro-Wilk tests). Numerical data were explored for normality. All showed normal (parametric) distribution except the (VAS) scores, which showed non-normal (non-parametric) distribution. Data were presented as median, range, mean, and standard deviation (SD) values. ANOVA test was used in a parametric repeated measures design to compare the three groups as well as to study the changes over time within each group. When the ANOVA test is significant, Bonferroni’s post-hoc test is used in pair-wise comparisons. The Kruskal-Wallis test was used to compare the non-parametric data of the three groups. Friedman’s test and the Wilcoxon signed-rank test were used to study the changes over time within each group. When Friedman’s test is significant, Dunn’s test is used in pair-wise comparisons. Qualitative data were presented as frequencies and percentages. (*P* ≤ 0.05) was the significance level.

## Results

### Baseline characteristics

At baseline, a non significant differences regarding baseline demographic or defect-related parameters were reported (*p* > 0.05). Groups I, II, and III comprised 57.1%, 57.1%, and 71.4% females, respectively, with mean ages of 36.1 ± 5.8, 37.1 ± 5.1, and 34.7 ± 5.0 years. Healing in all patients was uneventful, with no adverse events. In terms of site distribution, most defects were located in the mandible, representing 85.7%, 71.4%, and 85.7% in groups I, II, and III, respectively, while maxillary sites accounted for 14.3%, 28.6%, and 14.3%. Regarding tooth type, molars were the most frequently involved, comprising 85.7%, 71.4%, and 71.4%, followed by premolars (14.3%, 28.6%, and 28.6%) in the respective groups. Regarding defect morphology, the percentage of three-walled defects was 28.6%, 57.1%, and 57.1% in groups I, II, and III, respectively, where the percentage of two-walled defects was 71.4%, 42.9%, and 42.9% for the 3 groups. Table 1.

**Table 1:** Baseline characteristics of age, gender, tooth location, and intrabony defect morphology in the three groups

Baseline characteristics	Insulin + ACS (*n* = 7)	HA + ACS (*n* = 7)	ACS (*n* = 7)
Gender [n, (%)]			
Female	4 (57.1%)	4 (57.1%)	5 (71.4%)
Male	3 (42.9%)	3 (42.9%)	2 (28.6%)
Age [Mean, SD]	36.1 (5.8)	37.1 (5.1)	34.7 (5)
Site [n, (%)]			
Mandibular	6 (85.7%)	5 (71.4%)	6 (85.7%)
Maxillary	1 (14.3%)	2 (28.6%)	1 (14.3%)
Tooth [n, (%)]			
Premolar	1 (14.3%)	2 (28.6%)	2 (28.6%)
Molar	6 (85.7%)	5 (71.4%)	5 (71.4%)
Defect type [n, (%)]			
Two-osseous walls	5 (71.4%)	3 (42.9%)	3 (42.9%)
Three-osseous walls	2 (28.6%)	4 (57.1%)	4 (57.1%)

*Data are presented as mean ± SD or as frequency (%)

The result of the mean blood glucose level in the insulin group was (110.4 mg/dl) preoperatively and (121 mg/dl) postoperatively, with no significant difference postoperatively (*P* = 0.159).

### Clinical outcomes

Baseline CAL measurements were comparable among the insulin + ACS (9.14 ± 0.90 mm), HA + ACS (9.29 ± 0.95 mm), and ACS (8.57 ± 1.40 mm) groups (*P* = 0.457). Pair-wise intra-groups comparisons showed that CAL improved progressively within all groups at 6 months postoperative (*P* < 0.001; η² = 0.597–0.627). while intergroup differences were not statistically significant at any time point (baseline: *P* = 0.457; 6 months: *P* = 0.380) (Table 2). Additionally, baseline PPD measurements were comparable among the insulin + ACS (8.14 ± 0.90 mm), HA + ACS (8.14 ± 1.35 mm), and ACS (7.71 ± 0.95 mm) groups (*P* = 0.699). PPD decreased progressively in all groups, reaching 4.86 ± 1.35 mm, 5.71 ± 0.49 mm, and 4.86 ± 0.69 mm at 6 months for the insulin + ACS, HA + ACS, and ACS groups, respectively. Pair-wise comparisons showed that intragroup analysis showed significant time-dependent reductions in all groups (*P* < 0.001; η² = 0.63–0.69), while intergroup differences were not statistically significant at any time point (baseline: *P* = 0.699; 6 months: *P* = 0.159). Table 3.

**Table 2:** Descriptive statistics and results for comparison between CAL measurements (mm) in the three groups and the changes within each group

Time	Insulin + ACS (*n* = 7)		HA + ACS (*n* = 7)		ACS (*n* = 7)		*P*-value	Effect size (Partial Eta Squared)
	Mean	SD	Mean	SD	Mean	SD		
Base line	9.14 ^A^	0.9	9.29 ^A^	0.95	8.57 ^A^	1.4	0.457	0.083
3 months	7 ^B^	0	6.86 ^B^	1.77	6.57 ^B^	1.4	0.824	0.021
6 months	6.14 ^C^	1.46	6.86 ^B^	1.77	5.71 ^C^	1.25	0.38	0.102
*P*-value	< 0.001*		< 0.001*		< 0.001*			
*Effect size (Partial Eta Squared)*	0.623		0.627		0.597			

Data are presented as mean ± standard deviation (SD). Intergroup comparisons were performed using repeated measures ANOVA followed by Bonferroni’s post-hoc test. Different superscript letters (A, B, C) within the same column indicate statistically significant differences over time within each group (P ≤ 0.05)

**Table 3:** Descriptive statistics and results for comparison between PD measurements (mm) in the three groups and the changes within each group

Time	Insulin + ACS (*n* = 7)		HA + ACS (*n* = 7)		ACS (*n* = 7)		*P*-value	Effect size (Partial Eta Squared)
	Mean	SD	Mean	SD	Mean	SD		
Base line	8.14 ^A^	0.9	8.14 ^A^	1.35	7.71 ^A^	0.95	0.699	0.039
3 months	6 ^B^	0	5.71 ^B^	0.49	5.71 ^B^	0.95	0.615	0.053
6 months	4.86 ^C^	1.35	5.71 ^B^	0.49	4.86 ^C^	0.69	0.159	0.185
*P*-value	< 0.001*		< 0.001*		< 0.001*			
*Effect size (Partial Eta Squared)*	0.69		0.633		0.63			

Data are presented as mean ± standard deviation (SD). Intergroup comparisons were performed using repeated measures ANOVA followed by Bonferroni’s post-hoc test. Different superscript letters (A, B, C) within the same column indicate statistically significant differences over time within each group (P ≤ 0.05)

### Pain assessment

In all groups, VAS scores significantly decreased over time. After 24 h, Pair-wise comparisons revealed a non-significant difference between insulin (1.29 ± 0.49) and HA (1.43 ± 0.53) groups; both showed significantly lower pain scores than the control group (2.57 ± 0.53) (*p* = 0.004, η² = 0.589). At 48 h, further reductions were observed (*p* = 0.007, η² = 0.556), with insulin maintaining the lowest scores (0.29 ± 0.49). Pair-wise comparisons revealed a non-significant difference between the HA and control groups; both showed statistically significantly higher pain scores than the Insulin group. There was significant improvement over time (*p* < 0.05) in all groups. Table 4.

**Table 4:** Descriptive statistics for comparison between pain (VAS) scores in the three groups and changes within each group

Time	Insulin + ACS (*n* = 7)		HA + ACS (*n* = 7)		ACS (*n* = 7)		*P*-value	Effect size (Eta Squared)
	Median (Range)	Mean (SD)	Median (Range)	Mean (SD)	Median (Range)	Mean (SD)		
24 h	1 (1, 2) ^B^	1.29 (0.49)	1 (1, 2) ^B^	1.43 (0.53)	3 (2, 3) ^A^	2.57 (0.53)	0.004*	0.589
48 h	0 (0, 1) ^B^	0.29 (0.49)	1 (0, 1) ^A^	0.71 (0.49)	1 (1, 2) ^A^	1.43 (0.53)	0.007*	0.556
*P*-value	0.008*		0.025*		0.011*			
*Effect size (d)*	1		0.845		0.956			

Data are presented as median (range) and mean ± standard deviation. Intergroup comparisons were performed using Kruskal–Wallis test followed by Dunn’s post-hoc test. Different superscript letters (A, B) within the same row indicate statistically significant differences between groups at the same time point (P ≤ 0.05)

### Radiographic outcome

#### Radiographic linear defect depth (RLDD)

All groups demonstrated significant time-dependent improvement (*P* < 0.001; η² = 0.65–0.84). After 6 months, RLDD decreased to 6.04 ± 0.45 mm, 5.09 ± 0.66 mm, and 5.44 ± 0.68 mm in insulin, HA, and control groups, respectively, compared with baseline values of 8.10 ± 0.59 mm, 7.77 ± 0.54 mm, and 7.09 ± 0.55 mm. At baseline, Pair-wise comparisons revealed a non-significant difference between the insulin and HA groups; both showed significantly higher mean defect depth than the control group. A significant difference between groups was observed at 6 months (*P* = 0.027; η² = 0.332). Pair-wise comparisons revealed that HA and ACS groups showed greater defect depth reduction values than the Insulin group, while no significant difference was found between HÀ and control groups. Table 5.

**Table 5:** Descriptive statistics and results for comparison between radiographic linear defect depth (mm) in the three groups and the changes within each group

Time	Insulin + ACS (*n* = 7)		HA + ACS (*n* = 7)		ACS (*n* = 7)		*P*-value	Effect size (Partial Eta Squared)
	Mean	SD	Mean	SD	Mean	SD		
Base line	8.1 ^A^	0.59	7.77 ^A^	0.54	7.09 ^B^	0.55	0.010*	0.401
6 months	6.04 ^A^	0.45	5.09 ^B^	0.66	5.44 ^B^	0.68	0.027*	0.332
*P*-value	< 0.001*		< 0.001*		< 0.001*			
*Effect size (Partial Eta Squared)*	0.748		0.835		0.654			

Data are presented as mean ± standard deviation (SD). Intergroup comparisons were performed using repeated measures ANOVA followed by Bonferroni’s post-hoc test. Different superscript letters (A, B) within the same row indicate statistically significant differences between groups at the same time point (P ≤ 0.05)

#### Bone density (HU)

Preoperatively, all groups showed comparable mean densities (*p* = 0.499, η² = 0.074). Postoperatively, both insulin + ACS (164.41 ± 28.58 HU) and HA + ACS (163.57 ± 36.77 HU) demonstrated improvement compared to ACS (135.43 ± 34.45 HU) (*p* = 0.21, η² = 0.159). Within-group comparisons revealed significant increases in bone density for insulin + ACS (*p* < 0.001, η² = 0.753) and HA + ACS (*p* = 0.022, η² = 0.257), while the ACS group showed no significant change (*p* = 0.86, η² = 0.002). Table 6.

**Table 6:** Descriptive statistics for comparison between bone density (HU) in the three groups and the changes within each group

Time	Insulin + ACS (*n* = 7)		HA + ACS (*n* = 7)		ACS (*n* = 7)		*P*-value	Effect size (Partial Eta Squared)
	Mean	SD	Mean	SD	Mean	SD		
Pre-operative	133.73	33.65	153.23	35.99	136.17	29.26	0.499	0.074
Post-operative	164.41	28.58	163.57	36.77	135.43	34.45	0.21	0.159
*P*-value	< 0.001*		0.022*		0.86			
*Effect size (Partial Eta Squared)*	0.753		0.257		0.002			

Data are presented as mean ± standard deviation (SD). Asterisks (*) indicate statistically significant changes within groups over time (P ≤ 0.05)

## Discussion

Biological mediators within the local microenvironment are a pivotal determinant of periodontal regenerative outcomes, particularly when combined with minimally invasive surgical techniques. Further, the topical administration that integrates osteoconductive and osteoinductive biomaterials is an efficient option in this regard [3, 4]. This work presents insulin as a novel biomaterial for the management of periodontal intra-bony defects, as promising results have been shown. Topical insulin application was extensively investigated for its role in tissue repair, including wound healing [30], ulcer management [31], bone healing and regeneration [7, 32], and enhancement of implant osseointegration [10]. Despite these promising findings, research evaluating the regenerative potential of insulin in the management of periodontitis remains constrained.

Hyaluronic acid, an abundant component of the periodontal extracellular matrix, is found in higher concentrations in non-mineralized tissues than in mineralized structures. Extensive clinical and experimental evidence supported the adjunctive use of HA in periodontal treatment due to multifunctional biological effects like modulation of inflammation, promotion of angiogenesis, and facilitation of fibroblast and osteoblast migration [33]. In this work, we didn’t prescribe postoperative analgesics due to the generally reported low pain perception after different periodontal surgeries [34]. This coincides with the relatively low postoperative VAS scores reported in all study groups, especially since we employed the minimally invasive approach in our work [35]. Postoperative pain was significantly decreased in both the insulin and HA-treated groups after 24 h, compared to controls, whereas after 48 h, the insulin-treated group showed almost no postoperative pain compared to the other two groups. Both treatment modalities have valuable anti-inflammatory effects. Insulin markedly reduces the pro-inflammatory cytokine production (TNF-α and IL-6) and reduces oxidative stress responses [36]. Further, local insulin at the wound site enhances the neurite outgrowth, nerve repair, and microcirculation, which diminishes local inflammation [37]. Moreover, the known anti-inflammatory, bacteriostatic, antioxidant, and anti-edematous effects of HA contribute to a favorable wound healing environment [38].

This is the first pilot randomized clinical trial evaluating the regenerative efficacy of locally applied insulin versus HA, combined with an absorbable collagen sponge, in the management of periodontal intrabony defects using a single flap approach. The SFA was selected based on evidence supporting its predictability for managing intrabony defects with a vertical component of ≥ 3 mm [39]. Compared to other surgical techniques, SFA improves optimal functional and aesthetic outcomes through flap repositioning and suturing without detaching the papilla, reducing contamination risk, minimizing postoperative recession, and enhancing primary closure [40].

This work revealed clinical and radiographic healing enhancement in all treatment groups, with superiority of the intervention groups (insulin + ACS, and HA + ACS), compared to the control. The stability of blood glucose levels reported after treatment negates the systemic effect of locally applied insulin [41].

Clinically, a significant improvement in CAL and PPD was demonstrated postoperatively in all treatment modalities, with the most considerable improvements shown in the insulin group, indicating enhanced soft tissue regeneration and new attachment formation. In wound healing, insulin promotes fibroblast proliferation, collagen synthesis, angiogenesis, and accelerates epithelialization through modulation of inflammation and growth factors [42, 43].

The potential direct effects of local insulin delivery on bone healing have been investigated in the BB Wistar femur fracture model. Insulin delivery at the fracture site normalized the early (cellular proliferation and chondrogenesis) and late (mineralized tissue, cartilage content, and mechanical strength) parameters of diabetic fracture healing without affecting the systemic parameters of blood glucose [44]. These biological mechanisms likely underpin the favorable clinical outcomes observed in this work. Renu Devi et al. reported similar osteogenic effects with PPD reduction and CAL gain with insulin-like growth factor-1(IGF-I) therapy. IGF shares partial signaling pathways with insulin and exhibits strong bone regenerative potential [45]. The clinical improvement in the HA + ACS group can be attributed to the established HA bioactive effects, being an essential extracellular matrix component that promotes cell adhesion, migration, and cytokine modulation. HA also maintains tissue hydration, scavenges reactive oxygen species, and modulates inflammatory cell recruitment. All of these effects can support periodontal wound healing [46, 47].

In both test groups, the ACS is a biocompatible scaffold for space provision, clot stabilization, and cellular infiltration, thus amplifying the effect of locally applied agents [20]. Collagen sponges are well-characterized carrier systems that provide a sustained release of biomolecules with a putative role in bone regeneration [48]. The structural element of collagen could form a scaffold for cell interaction that facilitates cell adhesion, proliferation, and the degradation of the collagen scaffold could be absorbed by cells to promote the development of new tissue [49, 50]. In this work, the absence of postoperative adverse events could support the employed treatment modalities.

Radiographic findings revealed improvement in all tested parameters (radiographic linear defect depth and bone density*)*within all studied groups, showing the beneficial effect of the SFA with space maintenance in the management of intrabony defects. This is consistent with earlier reported data [20]. A non-significant difference was reported postoperatively between the three groups regarding bone density. Previous studies showed that insulin increased osteoblast differentiation and osteoclastogenesis [51]. Further, HA enhances osteoblast differentiation, increases mineralization, and bone formation, especially if mixed with bone grafts [52, 53]. It’s worth mentioning that our employed treatment of resorbable collagen sponge soaked with HA was earlier tested in an animal model, and this mix resulted in new bone formation in critical-size defects. However, the complete closure of the defects was not achieved, and healing in a considerable portion of the defects was characterized by fibrous tissue formation [48]. In the healing of the extraction socket, HA was effective in bone formation with enhanced expression of bone morphogenic protein and osteopontin, thus our results could be supported by such previous findings [54].

The slightly higher reduction of RLDD among the HA group and the controls compared to the insulin group may be explained by the prevalence of the three-wall defects in these groups, unlike the insulin group, which included more two-wall defects. The three-wall defects provide superior vascularity and regenerative osteogenic potential. However, the insulin group achieved good healing, supporting insulin’s potent regenerative influence on bone repair [55, 56]. Topical application of insulin, especially in non-diabetic clinical bone defects, has not received much attention. In this work, the insulin group demonstrated improvement in bone density, suggesting enhanced mineral maturation and remodeling. Insulin was reported to effectively promote local skull bone formation by increasing the number of osteoblasts and osteoid surface area in the mouse skull, and can regulate the activity of osteoclasts [7]. Conversely, limited radiographic increase was shown in the ACS-alone group, declaring the importance of bioactive adjuncts to collagen scaffold in optimization of periodontal regeneration [57].

Although this work showed promising outcomes related to the use of topical insulin in periodontal regeneration, the topical application of insulin in the healing of soft and bone tissues has challenges that should be highlighted in the context of the current work. Insulin has a short life, being liable to local degradation by high protease activity in the wound microenvironment. Moreover, topical conventional formulations have poor bioavailability and quick removal from moist wound environments, all plus the side effects of too much insulin. The half-life of free insulin is only 20–30 min [7, 58]. Because of the previously mentioned, we used long-acting insulin, although recent work reported no difference between using short versus long-acting insulin in the effective treatment of diabetic foot ulcer [59]. Thus, we can postulate that despite the limited stability and therapeutic efficacy of topically applied insulin, considerable beneficial effects were attained.

### Limitations and future perspectives

Although our results are promising, more studies are needed with a larger sample size. To validate true periodontal regeneration and assess the long-term stability of insulin-mediated bone formation, larger-scale investigations combined with histologic evaluation are required. Further, despite insulin capacity for periodontal regeneration, the development of new, more resilient, and bio-responsive drug delivery systems that can adjust to the changing wound environment to improve insulin local delivery and retention in the local environment is required.

## Conclusion

Within the limitations of this study, both insulin + ACS and HA + ACS combinations caused favorable clinical and radiographic regenerative outcomes compared to ACS alone. More work is needed to validate these findings.

## Data Availability

Data available on request due to privacy/ethical restrictions.
